# Supplementary material for: Quality of life of breast cancer patients in Amhara region, Ethiopia: A cross-sectional study
Source: PLoS One. 2024 Jun 27;19(6):e0305263. doi: 10.1371/journal.pone.0305263 (PMC11210875; doi:10.1371/journal.pone.0305263)
Supplement: S1 File — (DOCX) [file pone.0305263.s001.docx]

*ባህር ዳር ዩኒቨርስቲ ህክምናና ጤና ሳይንስ ኮሌጅ፤ የሕብረተሰብ ጤና አጠባበቅ ት/ቤት*

*ስነ-ተዋልዶ ጤናና ስነ ሕዝብ ትምህርት ክፍል*

*አባሪ* III*: -* አማረኛ ቃለ- መጠይቅ

**ሆስፒታል** 1. ፈለገ ህይወት 2. ጎንደር 3. ደሴ

**መለያ ቁጥር** ___________
የመረጃ ሰብሳቢው ስም ______________________ ቀን __________________ ፊርማ ___________

*ለበለጠ መረጃ፡ ታምራት ዓለም, ስልክ ቁጥር +251918606851, ኢ.ሜይል* [tamratalem34@gmail.com](mailto:tamratalem34@gmail.com)

**ክፍል 1፣ ግለሰባዊ መረጃዎች**

| ተ.ቁ | ጥያቄ | ምላሽ | ዝለል |
| --- | --- | --- | --- |
| 101. | ዕድሜ(ዓመት) ­­­­ | ___________ |  |
| 102. | የመኖርያ አድራሻ | 1. ከተማ 2. ገጠር |  |
| 103. | የጋብቻ ሁኔታ | 1.ያላገባች 3. በፍቺ ተለያይታ የምትኖር  2. ያገባች 4. ባሏ የሞተባት |  |
| 104. | የትምህርት ደረጃ | 1. መደበኛ ትምህርት ያልገባች  2. የመጀመርያ ደረጃ(1-8)  3. ሁለተኛ ደረጃ (9-12)  4. ዲፕሎማ  5. የመጀመርያ ድግሪ እና ከዚያን በላይ |  |
| 105. | ሀይማኖት | 1. ኦርቶዶክስ 3. ሙስሊም  2. ፕሮቴስታንት 4.ካቶሊክ 5. ሌላ_________ |  |
| 106. | ሥራ | 1. የቤት እመቤት 4. ተማሪ  2. የመንግሥት ሠራተኛ 5. የቀን ሠራተኛ  3. ነጋዴ 6. ሌላ(ግለፅ) ____________ |  |
| 107. | ለመጀመሪያ ጊዜ የጡት ካንሰር እንዳለብዎት የተነገረዎት መቼ ነው? | ______ ዓመት ____________ ወር |  |
| 108. | አሁን ላይ ያለው የጡት ካንሰሩ ደረጃ | 1. ደረጃ 1 2. ደረጃ 2 3. ደረጃ 3 4. ደረጃ 4 |  |
| 109. | ምን ዓይነት ህክምና ነው የተደረገልዎት? ከአንድ ምርጫ በላይ መምረጥ ይቻላን | 1. ኬሞቴራፒ 4. በቀጠሮ ተመላላሽ ታካሚ  2. የጨረር ህክምና 5. ሌላ(ገለፅ)_______________  3. ቀዶ ጥገና |  |
| 110. | ጥያቄ ቁ. 110 መልስዎ ቀዶ ጥገና ከሆነ፣ ምን አይነት ቀዶ ጥገና? | 1. ሙሉ ለሙሉ የጡት መቆረጥ  2. መጠነኛ ቀዶ ጥገና |  |
| 111. | ጥያቄ ቁ.110 መልስዎ ኬሞቴራፒ ከሆነ ስንተኛ ዙር ነው? | ____________ |  |
| 112. | የህክምና ወጭ በማን ነው ሚሸፈነው? | 1. በግል  2. ጤና መድን  3. ሌላ(ግለፅ)______ |  |

**ክፍል 2:- የሀብት ሁኔታን የሚመለከት ጥያቄ(ለከተማ ነዋሪወች ብቻ)**

| ተ.ቁ | ጥያቄ | ምላሽ |
| --- | --- | --- |
| 201. | የቤቱ ይዞታ የማን ነዉ? | 1. የግል 3. የክራይ  2.የቀበሌ 4.ሌላ______ |
| 202. | የቤቱ ወለል | 1. አፈር/ጠጠር 3. ሲሚንቶ  2. በዕበት የተለቀለቀ 4. ሴራሚክ |
| 203. | የቤቱ ጣራ | 1. ቆርቆሮ 2. ሳር 3. ሌላ ….. |
| 204. | የቤቱ የውጭ ግድግዳ | 1. ድንጋይ በጭቃ 3. ድንጋይ/ብሎኬት በስሚነቶ  2. ዕንጨት በጭቃ 4. ሌላ ______ |
| 205. | ለመኝታ የሚያገለግል ክፍል ብዛት | በቁጥር ______ |
| 206. | የቤቱ ዋና የምግብ ማብሰያ | 1. ኤሌክትሪክ 4. ኩበት  2. ከሰል 5. ጋዝ  3. እንጨት 6. ሌላ______ |
| 207. | የሚጠቀሙት መጸዳጃ ቤት ምን ዐይነት ነው | 1. የውሃ መልቀቂያና ማፋሰሻ ያለው የግል 4. የጋራ  2.የተሻሻል ሽታ አልባ የጉድጓ 5. ሜዳ ላይ  3. ባህላዊ የጉድገጓድ |
| 208. | የምግብ ማብሰያ ቤት አልዎት | 1.አወ 2.የለም |
| 209. | የሚጠቀሙበት የውኃ ምንጭ ምንድን ነው | 1. በግቢው ውስጥ ካለ ቧንቧ 5. የጉድጓድ ውኃ ፓምፕ ያለው  2. የጋራ ጉድጓድ 6. የተጠራቀመ የዝናብ ውኃ  3.የጋራ ቧንቧ 7.ኩሬ/ወንዝ/ወራጅ/ምንጭ  4.ግቢዎ ውስጥ የጉድጓድ ውኃ ከነፓንፑ 8. የጉድጓድ ውኃ ፓምፕ የሌለው |
| 210. | ቤት ውስጥ ከዚህ በታች የተዘረዘሩት አሉ? አወ ካሉ ቁጥራቸው ምን ያህል ነው | |
|  | ሬዲዮ | 1. አወ 2.የለም |
|  | ቴለተቪዥን | 1. አወ 2.የለም |
|  | የቤት ስልክ | 1. አወ 2.የለም |
|  | ፍሪጅ | 1. አወ 2.የለም |
|  | አልጋ /የጥጥ/ ስፖንጅ/ስፕሪንግፍራሽ | 1. አወ 2.የለም |
|  | ጠረጴዛ | 1. አወ 2.የለም |
|  | ወንበር | 1. አወ 2.የለም |
| 211 | ከቤተሰብዎ ውስጥ ከሚከተሉት ያለው አለ? መልስዎ አወ ከሆን ምን አይነት ነው | |
|  | የዕጅ ስልክ | 1. አወ 2. የለም |
|  | ሞተር ሳይክል | 1. አወ 2. የለም |
|  | ባጃጅ | 1. አወ 2. የለም |
|  | ጋሬ | 1. አወ 2. የለም |
|  | መኪና | 1. አወ 2. የለም |
|  | በሬ/ላም | 1. አወ 2. የለም |
|  | ፈረስ/በቅሎ | 1. አወ 2. የለም |
|  | ፍየል/በግ | 1. አወ 2. የለም |
|  | ደሮ | 1. አወ 2. የለም |
|  | የንብ ቀፎ | 1. አወ 2. የለም |
|  | የባንክ/የቁጠባ ደብተር | 1. አወ 2. የለም |

| ***ከገጠር ለመጡ ብቻ የሚሞላ*** | | |
| --- | --- | --- |
| ተ.ቁ | ጥያቄ | ምላሽ |
| 301. | የቤቱ ይዞታ የማን ነዉ? | 1. የግል 2.ሌላ ______ |
| 302. | የቤቱ ጣራ | 1. ቆርቆሮ 3. ሌላ ______  2. ሳር/ዕንጨት |
| 303. | የቤቱ የውጭ ግድግዳ | 1. ድንጋይ በጭቃ 3. ድንጋይ/ብሎኬት በስሚነቶ  2. ዕንጨት በጭቃ 4. ሌላ_____ |
| 304. | የቤቱ ዋና የምግብ ማብሰያ | 1. ኤሌክትሪክ 4. ኩበት  2. ከሰል 5. ጋዝ  3. እንጨት 6. ሌላ ______ |
| 305. | ለእንስሳቱ መኖሪያ የሚሆን የተለየ ቤት አልወት | 1. አወ 2. የለም |
| 306. | የምግብ ማብሰያ ቤት አልዎት | 1.አወ 2.የለም |
| 307. | የሚጠቀሙበት የውኃ ምንጭ ምንድን ነው | 1. በግቢው ውስጥ ካለ ቧንቧ 6.ኩሬ/ወንዝ/ወራጅ/ምንጭ  2. የጋራ ጉድጓድ 7. የጉድጓድ ውኃ ፓምፕ የሌለው  3.የጋራ ቧንቧ 8.የተገደበ ውሃ  4.ግቢዎ ውስጥ የጉድጓድ ውኃ ከነፓንፑ  5. የተጠራቀመ የዝናብ ውኃ 9. ሌላ ______ |
| 308. | ቤት ውስጥ ከዚህ በታች የተዘረዘሩት አሉ? አወ ካሉ ቁጥራቸው ምን ያህል ነው | |
| . | ሬዲዮ ወይም ቴፕ | 1. አወ 2.የለም |
|  | አልጋ/የጥጥ/ ስፖንጅ/ስፕሪንግፍራሽ | 1. አወ 2.የለም |
|  | የሞባይል ስልክ | 1. አወ 2.የለም |
|  | የውኃ ጄኔሬተር | 1. አወ 2.የለም |
|  | የሶላር ሀይል | 1. አወ 2.የለም |
|  | ሌላ ካለ ይጥቀሱ | ____________ |
| 309. | የቤት እንሰሳ | |
|  | በሬ/ላም | 1. አወ __________________ 2.የለም |
|  | ጥጃ | 1. አወ __________________ 2.የለም |
|  | አህያ | 1. አወ __________________ 2.የለም |
|  | ፈረስ/በቅሎ | 1. አወ __________________ 2.የለም |
|  | ፍየል/በግ | 1. አወ __________________ 2.የለም |
|  | ደሮ | 1. አወ __________________ 2.የለም |
|  | የንብ ቀፎ | 1. አወ __________________ 2.የለም |
| 310. | ከዚህ በታች የተጠቀሱት የእርሻና የጓሮ ምርት ባለፈው አመት(2010) አምርተዋል አወ ካሉ በኩንታል ምን ያህል አመረቱ | |
|  | ጤፍ | 1. አወ __________________ 2.የለም |
|  | ገብስ/ስንዴ | 1. አወ __________________ 2.የለም |
|  | በቆሎ | 1. አወ __________________ 2.የለም |
|  | ማሽላ | 1. አወ __________________ 2.የለም |
|  | ሰሊጥ | 1. አወ __________________ 2.የለም |
|  | ባቄላ/አተር/ሽንብራ | 1. አወ __________________ 2.የለም |
|  | ምስር | 1. አወ __________________ 2.የለም |
|  | ዳጉሳ | 1. አወ __________________ 2.የለም |
| 311. | ሌሎች ምርቶች ካሉ የጥቀሱ | |
|  | 1.___________________________ 2.____________________________ | 3.____________________________ 4.____________________________ |

**ክፍል** 2:-**EORTC QOL C-30 አማረኛ ቃለ- መጠይቅ**

እርስዎንና ጤንነትዎን በተመለከተ የተወሰኑ ነገሮችን ለማወቅ እንፈልጋለን። እባክዎትን የሚከተሉትን ጥያቄዎች በሙሉ እርስዎ ትክክለኛ ነው ብለው ያመኑበትን በማክበብ ይመልሱ። «ትክክለኛ» መልስ ወይም «የተሳሳተ» መልስ የሚባል የለም። የሚሰጡት መረጃ ሁሉ ምስጢራዊነቱ በደንብ የተጠበቀ ይሆናል። 1. በጭራሽ 2. በትንሹ 3. በመጠኑ 4. በጣም በብዛት

| 1. እንደ ከባድ ዘንቢል ወይም ሻንጣ መሸከም የመሳሰሉ ጉልበት የሚጠይቁ ስራዎችን ለመስራት ይቸገሩ ነበር? | በጭራሽ | በትንሹ | በመጠኑ | | በጣም በብዛት |
| --- | --- | --- | --- | --- | --- |
| 2. ረዥም የእግር ጉዞ ለማድረግ ችግር አለብዎት? | 1 | 2 | 3 | | 4 |
| 3. ከቤትዎ ውጭ አጭር የእግር ጉዞ ለማድረግ ችግር አለብዎት? | 1 | 2 | 3 | | 4 |
| 4. በህመምዎ የተነሳ በቀን አልጋ ወይም ወንበር ላይ ሆነው ረዘም ላለ ሰዓት ያሳልፋሉ? | 1 | 2 | 3 | | 4 |
| 5. ሲመገቡ፣ ሲለብሱ፣ ሲታጠቡ ወይም ሽንት ቤት ሲጠቀሙ እገዛ ያስፈልግዎታል? | 1 | 2 | 3 | | 4 |
| ባለፈው ሳምንት ውስጥ | | | | | |
| 6. ስራዎትን ወይንም የዕለት ተዕለት እንቅስቃሴዎትን ለማከናወን ይከብድዎት ነበር? | 1 | 2 | 3 | 4 | |
| 7. በትርፍ ጊዜ የሚከናወኑ ስራዎችን ወይንም ሌሎች የመዝናኛ ጊዜዎችን ለማሳለፍ ይከብድዎት ነበር? | 1 | 2 | 3 | 4 | |
| 8. ሲተነፍሱ የትንፋሽ ማጠር አጋጥሞት ነበር? | 1 | 2 | 3 | 4 | |
| 9. የህመም ስሜት ነበረብዎ? | 1 | 2 | 3 | 4 | |
| 10. ከወትሮው የተለየ ዕረፍት አስፈልጎዎት ነበር? | 1 | 2 | 3 | 4 | |
| 11. የዕንቅልፍ ችግር ነበረብዎ? | 1 | 2 | 3 | 4 | |
| 12. አቅም ያንስዎት ነበር? | 1 | 2 | 3 | 4 | |
| 13. የምግብ ፍላጎትዎ ቀንሷል? | 1 | 2 | 3 | 4 | |
| 14. የማቅለሽለሽ ስሜት ነበረብዎ? | 1 | 2 | 3 | 4 | |
| 15. አስመልሶዎት ነበር? | 1 | 2 | 3 | 4 | |
| 16. የሰገራ ድርቀት ነበረብዎ? | 1 | 2 | 3 | 4 | |
| 17. ተቅማጥ ነበረብዎ? | 1 | 2 | 3 | 4 | |
| 18. የድካም ስሜት ነበረዎ? | 1 | 2 | 3 | 4 | |
| 19. ህመሙ የዕለት ተዕለት እንቅስቃሴዎትን ያውክብዎ ነበር? | 1 | 2 | 3 | 4 | |
| 20. አንድ አንድ ነገሮችን ትኩረት ሰጥተው ለመስራት ያውክዎት ነበር (ለምሳሌ፤ ጋዜጣ ለማንበብ፣ ራዲዮ ለማዳመጥ)? | 1 | 2 | 3 | 4 | |
| 21. የውጥረት ስሜት ነበረብዎ? | 1 | 2 | 3 | 4 | |
| 22. የመጨነቅ ስሜት ነበረብዎ? | 1 | 2 | 3 | 4 | |
| 23. የመነጫነጭ ስሜት ነበረብዎ? | 1 | 2 | 3 | 4 | |
| 24. የመደበር ስሜት ነበረብዎ? | 1 | 2 | 3 | 4 | |
| 25. ነገሮችን የማስታወስ ችግር ነበረብዎ? | 1 | 2 | 3 | 4 | |
| 26. አካላዊ ሁኔታዎ ወይም የሚከታተሉት ህክምና በቤተሰባዊ ህይወትዎ ላይ ያሳደረው ተጽዕኖ ነበር? | 1 | 2 | 3 | 4 | |
| 27. የጤናዎ ሁኔታ ወይም የሚከታተሉት ህክምና በማህበራዊ ሕይወትዎ በሚደርጉት እንቅስቃሴዎ ላይ ያሳደረው ተጽዕኖ ነበር? | 1 | 2 | 3 | 4 | |
| 28. የጤናዎ ሁኔታ ወይም የሚከታተሉት ህክምና ገንዘብ እንዲያጥርዎ /እንዲቸግርዎ/ አድርጓል? | 1 | 2 | 3 | 4 | |

ለሚከተሉት ጥያቄዎች ከ1-7 ካሉት ቁጥሮች ውስጥ እርስዎን በደንብ የሚገልጽዎትን አንዱን ቁጥር ያክብቡ

29. በአጠቃላይ ባለፈው ሳምንት የነበረዎትን የጤንነት ሁኔታ እንዴት ይመዝኑታል?

1 2 3 4 5 6 7
በጣም መጥፎ እጅግ በጣምጥሩ
30. በአጠቃላይ ባለፈው ሳምንት የነበረዎትን የኑሮ ጥራት ሁኔታ እንዴት ይመዝኑታል?

1 2 3 4 5 6 7
በጣም መጥፎ እጅግ በጣምጥሩ

**ክፍል 3፡-EORTC QLQ - BR23**

እባክዎትን ባለፈው ሣምንት ውስጥ እርስዎን የገጠመዎትን የህምም ምልክት ወይም ችግር ምን ያህል እንድሆን ይግለፁ፡፡ 1. በጭራሽ 2. በትንሹ 3. በመጠኑ 4. በጣም በብዛት

| **ባለፉት ሳምንታት ውስጥ** | በጭራሽ | | በትንሹ | በመጠኑ | በጣም በብዛት |
| --- | --- | --- | --- | --- | --- |
| 31. አፍዎ ይደርቅብዎት ነበር? | **1** | | **2** | **3** | **4** |
| 32. የምግብና የመጠጥ ጣዕም ከበፊቱ ተለይቶብዎት ነበር ? | **1** | | **2** | **3** | **4** |
| 33. አይንዎትን የማመም፣ የመቆጥቆጥ ወይም እንባ የማቅረር ስሜት ነበረብዎት? | **1** | | **2** | **3** | **4** |
| 34. ፀጉርዎ ሳስቶ ወይም ተመልጦ ነበር?? | **1** | | **2** | **3** | **4** |
| 35. ይህንን ጥያቄ ፀጉርዎ ሳስቶ ወይም ተመልጦ ከነበረ ብቻ ይመልሱ፦ ፀጉርዎ በመሳሳቱ ወይም በመመለጡ ተበሳጭተው ነበር? | **1** | | **2** | **3** | **4** |
| 36. የህመም ወይም ጤነኛ ያለመሆን ስሜት ነበረብዎት? | **1** | | **2** | **3** | **4** |
| 37. ፊትዎ አካባቢ ድንገተኛ ሙቀት ማላብ እና ማቃጠል ተሰምቶዎት ነበር ? | **1** | | **2** | **3** | **4** |
| 38. ራስ ምታት ነበረብዎት? | **1** | | **2** | **3** | **4** |
| 39. በሕመምዎ ወይም በሕክምናው ምክንያት ዓይን የማይስቡ ሰው እንደሆኑ ዓይነት ስሜት ተሰምቶዎት ነበር? | **1** | | **2** | **3** | **4** |
| 40. በህመምዎ ወይም በህክምናው ምክንያት ሴትነትዎ እንደቀነሰብዎት ተሰምቶዎት ነበር? | **1** | | **2** | **3** | **4** |
| 41. ራቁት ሰውነትዎን ማየት ይቸግርዎት ነበር ? | **1** | | **2** | **3** | **4** |
| 42. በሰውነትዎ አቋም ያልረኩበት ጊዜ ነበር ? | **1** | | **2** | **3** | **4** |
| 43. ለወደፊት ጤንነትዎ ይጨነቁ ነበር ? | **1** | | **2** | **3** | **4** |
| **ባለፉት አራት ሳምንታት ውስጥ** | | | | | |
| 44. ለፆታዊ ግንኙነት ፍላጐትዎ ምን ያህል ነበር ? | **1** | | **2** | **3** | **4** |
| 45. ፆታዊግንኙነት ላይ ምን ያህል ተሳታፊ ነበሩ? (ከግብረስጋ ግንኙነት ጋር ወይም ካለ ግብረስጋ ግንኙነት) | **1** | | **2** | **3** | **4** |
| 46. ይህን ጥያቄ ፆታዊ ግንኙነት ላይ ተሳታፊ ከነበሩ ብቻ ይመልሱ፦ ፆታዊ ግንኙነቱ ለእርስዎ ምን ያህል አስደሳች ነበር? | **1** | | **2** | **3** | **4** |
| **ባለፉት ሳምንታት ውስጥ** | | | | | |
| 47. ክንድዎትን ወይም ትከሻዎትን ህመም ተሰምቶዎት ነበር ? | | **1** | **2** | **3** | **4** |
| 48. ክንድዎ ወይም እጅዎት አብጦ ነበር ? | | **1** | **2** | **3** | **4** |
| 49. ክንድዎትን ለማንሳት ወይም ወደ ጐን ለማንቀሳቀስ ይቸግሮት ነበር ? | | **1** | **2** | **3** | **4** |
| 50. በበሽታ በተጠቃው ጡትዎ አካባቢ ህመም ይሰማዎት ነበር ? | | **1** | **2** | **3** | **4** |
| 51. በበሽታ የተጠቃው ጡትዎ አካባቢ አብጦ ነበር ? | | **1** | **2** | **3** | **4** |
| 52. በበሽታ የተጠቃው ጡትዎ አካበቢ በትንሹ ሲነካ ከባድ ስሜት ነበረው? | | **1** | **2** | **3** | **4** |
| 53. በበሽታ በተጠቃው ጡትዎ አካባቢ የቆዳ ችግር ነበር (ምሳሌ፣ ማሳከክ፣ የመድረቅ፣ የ መላላጥ)? | | **1** | **2** | **3** | **4** |

**በጣም እናመሰግናለን!!!**
