# Supplementary material for: Quality of life of breast cancer patients in Amhara region, Ethiopia: A cross-sectional study
Source: PLoS One. 2024 Jun 27;19(6):e0305263. doi: 10.1371/journal.pone.0305263 (PMC11210875; doi:10.1371/journal.pone.0305263)
Supplement: S2 File — (DOCX) [file pone.0305263.s002.docx]

Annex I- English version Questionnaire

Hospitals 1. Felege Hiowt 2. Gondar 3. Dessie

Code _______ Name of Data collector _______________ Date__________ Signature _______.

For further Tamrat Alem Almaw, Phone+251918606851, Email: [tamratalem34@gmail.com](mailto:tamratalem34@gmail.com)

**PART I: - Socio-demographic data**

| S/N^o^ | Questions | Response | Skip |
| --- | --- | --- | --- |
| 101. | Age (years) | __________ |  |
| 102. | Place of residence | 1. Urban 2. Rural |  |
| 103. | Marital status | 1. Single 3. Divorced  2. Married 4. Windowed |  |
| 104. | Educational level | 1. No formal education 4. Diploma  2. Primary education (1-8) 5. Degree and above  3. Secondary education(9-12) |  |
| 106. | Religious | 1. Orthodox 3. Muslim  2. Protestant 4.catholic 5. Others |  |
| 107. | Occupational | 1. Housewife 4. Student  2. Government employee 5. Daily labor  3. Merchant 6. Others (specify) ---- |  |
| 108. | Time since diagnosis | __________years _______ months |  |
| 109. | Stage of disease | 1. Stage I 2. Stage II 3.stage III 4. stage IV |  |
| 110. | What type of treatment have you received? (You can circle more than one answer) | 1. Chemotherapy 4. Post- treatment follow up  2. Radiotherapy 5. Other (specify)________  3. Surgery |  |
| 111. | If Q110, the response is surgery, what type of surgery? | 1. Mastectomy  2. Conserving Surgery |  |
| 112. | If Q110, the response is chemotherapy, for how long? (cycle) | ___________________ |  |
| 113. | Cost of treatment | 1. Self/private  2. Health Insurance 3.Other________ |  |

| **Part II:- Wealth Indexes for Urban residence** | | | | |
| --- | --- | --- | --- | --- |
| S/N | Question | Response | | |
| 201. | Owner of the house | 1. Private 3. Rents  2. kebele 4. Other______ | | |
| 202. | House floor | 1. Soil 3. cement  2. Muck 4.ceramics | | |
| 203. | House roof | 1. Corrugated iron 2. Grass 3. Other ….. | | |
| 204. | Houses outside the wall | 1. Stone with mud 3. stone/brocket with cement  2. Wood with mud 4. Others ______ | | |
| 205. | Berth (how many) | In Number______ | | |
| 206. | Which of the following types of fuel does your household used mainly | 1. Electricity 4. Animal Dug  2. Charcoal 5. Gas  3. Wood 6. Others______ | | |
| 207. | What types of toilet do your household use? | 1. Flush or pour-flush latrine dwelling 4. Public latrine  2. Ventilated improved pit latrine 5. Space field  3. Traditional latrine | | |
| 208. | Kitchen house | 1.Yes 2.No | | |
| 209. | Source of water | 1. Piped water into the compound 2. Public Borehole  3. Public tap 4.Borehole with hand pump into the compound  5. Dam from rain source 6. pond/river/race/head  7. Borehole without hand pump into the compound  8. Dam from the river source | | |
| 210 | Do any of your household members have the following? If yes how many? | | | |
|  | Radio | | 1. Yes 2. No | |
|  | Television | | 1. Yes 2. No | |
|  | non-mobile telephone | | 1. Yes 2. No | |
|  | Refrigerator | | 1. Yes 2. No | |
|  | Bed/cotton/sponge/spring matters | | 1. Yes 2. No | |
|  | Table | | 1. Yes __________________ 2. No | |
|  | Chair | | 1. Yes __________________ 2. No | |
| 211 | Do any of your household members have the following? | | | |
|  | Mobile Phone | | | 1. Yes 2. No |
|  | Motor Cycle | | | 1. Yes 2. No |
|  | Bajaj | | | 1. Yes 2. No |
|  | Animal draw cart | | | 1. Yes 2. No |
|  | Car or truck | | | 1. Yes 2. No |
|  | oxen/cow | | | 1. Yes 2. No |
|  | Horse/mule | | | 1. Yes 2. No |
|  | Goat/Sheep | | | 1. Yes 2. No |
|  | Hen | | | 1. Yes 2. No |
|  | Beehives | | | 1. Yes 2. No |
|  | Bank account/ Saving Book | | | 1. Yes 2. No |

| ***Only for rural residence*** | | | |
| --- | --- | --- | --- |
| S/N | Question | Response | |
| 301 | Owner of the house | 1. Private 2. Others ______ | |
| 302 | House roof | 1. Corrugated iron 2. Grass/wood 3. Others ______ | |
| 303 | House outside wall | 1. Stone with mud 3. stone/blocket with cement  2. Wood with mud 4. Others___ | |
| 304 | The main source of houses’ cooking | 1. Electricity 4. Animal Dug 5. Gas  2. Charcoal 3. Wood 6 Others______ | |
| 305 | Does the household own separate house for domestic animals | 1. Yes 2. No | |
| 306 | Kitchen house | 1 Yes 2. No | |
| 307 | Source of water | 1. Piped water into the compound 5. Dam from rain source  2. public Borehole 6.pond/river/race/headwaters  3 Public tap 8. Dam from the river source  4. Borehole with hand pump into the compound  7. Borehole without hand pump into the compound | |
| 308 | Does any member of your household have the following? If yes how many? | | |
|  | Radio or Tape | | 1. Yes 2. No |
|  | Bed/ cotton/ sponge/spring/ mattress | | 1. Yes 2. No |
|  | Mobile Phone | | 1. Yes 2. No |
|  | Water generator | | 1. Yes 2. No |
|  | Solar energy | | 1. Yes 2. No |
|  | Other(specify) ________________ | |  |
| 310 | Domesticated animals | | |
|  | Ox/cow | | 1. Yes ______________ 2.No |
|  | Calf | | 1. Yes ______________ 2.No |
|  | Donkey | | 1. Yes ______________ 2.No |
|  | horse/mule | | 1. Yes ______________ 2.No |
|  | Goat/sheep | | 1. Yes ______________ 2.No |
|  | Hen | | 1. Yes _______________ 2.No |
|  | Beehives | | 1. Yes ______________ 2.No |
| 311 | Does the following agricultural production products in the last year(2018)? If yes how much in quintal? | | |
|  | Teff | | 1. Yes _______________ 2.No |
|  | Barley/Wheat | | 1. Yes _______________ 2.No |
|  | Maize | | 1. Yes _______________ 2.No |
|  | Millet | | 1. Yes ________________ 2.No |
|  | Sesame Seed | | 1. Yes ________________ 2.No |
|  | Bean/Pea | | 1. Yes _________________ 2.No |
|  | Chickpea | | 1. Yes _________________ 2.No |
|  | Lentil | | 1. Yes _________________ 2.No |
|  | Dagusa | | 1. Yes _________________ 2.No |
| 312 | If there are other productions (list) | |  |
|  | 1.____________________________ 2.____________________________ | | 3.____________________________ 4.____________________________ |

**PART II- The QLQ-C30 Version 4**

We are interested in some things about you and your health. Please answer all of the questions yourself by circling the number that best applies to you. There is no "right" or "wrong" answers. The information that you provide will remain strictly confidential.

1=Not at all, 2=A little, 3=Quite a bit,4= Very much

| Questions | Not at all | A little | Quite a bit | Very much |
| --- | --- | --- | --- | --- |
| 1. Do you have any trouble doing strenuous activities, like carrying a heavy shopping bag or a suitcase? | 1 | 2 | 3 | 4 |
| 2. Do you have any trouble taking a long walk? | 1 | 2 | 3 | 4 |
| 3. Do you have any trouble taking a short walk outside of the house? | 1 | 2 | 3 | 4 |
| 4. Do you need to stay in bed or a chair during the day? | 1 | 2 | 3 | 4 |
| 5. Do you need help with eating, dressing, washing yourself or using the toilet? | 1 | 2 | 3 | 4 |
| **During the past week** |  |  |  |  |
| 6. Were you limited in doing either your work or other daily activities? | 1 | 2 | 3 | 4 |
| 7. Were you limited in pursuing your hobbies or other | 1 | 2 | 3 | 4 |
| 8. Were you short of breath? | 1 | 2 | 3 | 4 |
| 9. Have you had pain? | 1 | 2 | 3 | 4 |
| 10. Did you need to rest? | 1 | 2 | 3 | 4 |
| 11. Have you had trouble sleeping? | 1 | 2 | 3 | 4 |
| 12. Have you felt weak? | 1 | 2 | 3 | 4 |
| 13. Have you lacked appetite? | 1 | 2 | 3 | 4 |
| 14. Have you felt nauseated? | 1 | 2 | 3 | 4 |
| 15. Have you vomited? | 1 | 2 | 3 | 4 |
| 16. Have you been constipated? | 1 | 2 | 3 | 4 |
| 17. Have you had diarrhea? | 1 | 2 | 3 | 4 |
| 18 Were you tired? | 1 | 2 | 3 | 4 |
| 19. Did pain interfere with your daily activities? | 1 | 2 | 3 | 4 |
| 20. Have you had difficulty in concentrating on things, like reading a newspaper or watching television? | 1 | 2 | 3 | 4 |
| 21. Did you feel tense? | 1 | 2 | 3 | 4 |
| 22. Did you worry? | 1 | 2 | 3 | 4 |
| 23. Did you feel irritable? | 1 | 2 | 3 | 4 |
| 24. Did you feel depressed? | 1 | 2 | 3 | 4 |
| 25. Have you had difficulty remembering things? | 1 | 2 | 3 | 4 |
| 26. Has your physical condition or medical treatment interfered with your family life? | 1 | 2 | 3 | 4 |
| 27. Has your physical condition or medical treatment interfered with your social activities? | 1 | 2 | 3 | 4 |
| 28. Has your physical condition or medical treatment caused your financial difficulties? | 1 | 2 | 3 | 4 |

For the following questions please choose the number between 1 and 7 that best apply to you
29. How would you rate your overall health during the past week?

1 2 3 4 5 6 7

Very poor Excellent

30. How would you rate your overall quality of life during the past week?

1 2 3 4 5 6 7

Very poor Excellent

**Part III- EORTC QOL - BR23**

Patients sometimes report that they have the following symptoms or problems. Please indicate the extent to which you have experienced these symptoms or problems during the past week. 1=Not at all, 2=A little, 3=Quite a bit, 4= Very much

| Questions | Not at all | A little | Quite a bit | Very much |
| --- | --- | --- | --- | --- |
| **During the past week** | | | | |
| 31. Did you have a dry mouth? | 1 | 2 | 3 | 4 |
| 32. Did food and drink taste different than usual? | 1 | 2 | 3 | 4 |
| 33. Were your eyes painful, irritated or watery? | 1 | 2 | 3 | 4 |
| 34. Have you lost any hair? | 1 | 2 | 3 | 4 |
| 35. Answer this question only if you had any hair loss: Were you upset by the loss of your hair? | 1 | 2 | 3 | 4 |
| 36. Did you feel ill or unwell? | 1 | 2 | 3 | 4 |
| 37. Did you have hot flushes? | 1 | 2 | 3 | 4 |
| 38. Did you have headaches? | 1 | 2 | 3 | 4 |
| 39. Have you felt physically less attractive as a result of your disease or treatment? | 1 | 2 | 3 | 4 |
| 40. Have you been feeling less feminine as a result of your disease or treatment? | 1 | 2 | 3 | 4 |
| 41. Did you find it difficult to look at yourself naked? | 1 | 2 | 3 | 4 |
| 42. Have you been dissatisfied with your body? | 1 | 2 | 3 | 4 |
| 43. Were you worried about your health in the future? | 1 | 2 | 3 | 4 |
| **During the past four week** | | | | |
| 44. To what extent were you interested in sex? | 1 | 2 | 3 | 4 |
| 45. To what extent were you sexually active? (with or without intercourse) | 1 | 2 | 3 | 4 |
| 46. Answer this question only if you have been sexually active: To what extent was sex enjoyable for you? | 1 | 2 | 3 | 4 |
| 47. Did you have any pain in your arm or shoulder? | 1 | 2 | 3 | 4 |
| **During the past week** | | | | |
| 48. Did you have a swollen arm or hand? | 1 | 2 | 3 | 4 |
| 49. Was it difficult to raise your arm or to move it sideways? | 1 | 2 | 3 | 4 |
| 50. Have you had any pain in the area of your affected breast? | 1 | 2 | 3 | 4 |
| 51. Was the area of your affected breast swollen? | 1 | 2 | 3 | 4 |
| 52. Was the area of your affected breast oversensitive? | 1 | 2 | 3 | 4 |
| 53. Have you had skin problems on or in the area of your affected breast (e.g., itchy, dry, flaky)? | 1 | 2 | 3 | 4 |

**Thank you very much for your help!**
