# Supplementary material for: Quality of life of breast cancer patients in Amhara region, Ethiopia: A cross-sectional study
Source: PLoS One. 2024 Jun 27;19(6):e0305263. doi: 10.1371/journal.pone.0305263 (PMC11210875; doi:10.1371/journal.pone.0305263)
Supplement: Attachment — Submitted filename: Response to Reviewers.docx [file pone.0305263.s005.docx]

**Response to academic editor**

**Comment**: Language revision of the manuscript.

***Response:*** *we thank you very much****.*** *We tried to edit the entire document of the manuscript. We edited the spelling, space between words, and the whole grammar as much as possible with online grammar and language checkers (Quill bot online checker).*

**Response to reviewers**

**Response to reviewer #2**

**Comment #1:** If the authors have adequately addressed your comments raised in a previous round of review and you feel that this manuscript is now acceptable for publication, you may indicate that here to bypass the “Comments to the Author” section, enter your conflict of interest statement in the “Confidential to Editor” section, and submit your "Accept" recommendation.

***Response #1****: Thank you very much.*

**Comment #2:** Is the manuscript technically sound, and do the data support the conclusions?
The manuscript must describe a technically sound piece of scientific research with data that supports the conclusions. Experiments must have been conducted rigorously, with appropriate controls, replication, and sample sizes. The conclusions must be drawn appropriately based on the data presented.

***Response #2****: Thank you very much for taking some spare time to review our paper.*

**Comment #3:** Has the statistical analysis been performed appropriately and rigorously? ***Response #3:*** *Thank you very much for taking some spare time to review our paper.*

**Comment #4:** Have the authors made all data underlying the findings in their manuscript fully available?
The PLOS Data policy requires authors to make all data underlying the findings described in their manuscript fully available without restriction, with rare exception (please refer to the Data Availability Statement in the manuscript PDF file). The data should be provided as part of the manuscript or its supporting information, or deposited to a public repository. For example, in addition to summary statistics, the data points behind means, medians and variance measures should be available. If there are restrictions on publicly sharing data—e.g. participant privacy or use of data from a third party—those must be specified.

***Response #4:*** *Thank you very much. We uploaded the study’s minimal underlying dataset as Supporting Information files.*

**Comment #5:** Is the manuscript presented in an intelligible fashion and written in standard English?
PLOS ONE does not copyedit accepted manuscripts, so the language in submitted articles must be clear, correct, and unambiguous. Any typographical or grammatical errors should be corrected at revision, so please note any specific errors here.

***Response #5:*** *Thank you very much for taking some spare time to review our paper.*

**Comment #6**: Review Comments to the Author
Please use the space provided to explain your answers to the questions above. You may also include additional comments for the author, including concerns about dual publication, research ethics, or publication ethics. (Please upload your review as an attachment if it exceeds 20,000 characters)

**Response #6:** *Thank you very much. We have revised the whole manuscript for dual publication and research ethics.*
