# Supplementary material for: Quality of life of breast cancer patients in Amhara region, Ethiopia: A cross-sectional study
Source: PLoS One. 2024 Jun 27;19(6):e0305263. doi: 10.1371/journal.pone.0305263 (PMC11210875; doi:10.1371/journal.pone.0305263)
Supplement: Attachment — Submitted filename: Response to reviewer.docx [file pone.0305263.s006.docx]

**Response to reviewer**

**Response to reviewer #3**

**Comment #1:** Line 112, the sample size calculation, how does 248 come from? Based on the given information, n should be 246. Considering the 10% non-response rate, the final sample size 273 should be obtained by 246/0.9=273.

***Response #1:*** *Thank for the insightful comment. The reviewer concern is right and meaningful. The error happened because of editorial mistake, but it has no effect on the final sample size. We corrected it in this revision.*

**Comment #2:** Line 207, there are only 256 patients with a response rate of 93.4%. Please explain why the recruitment of patients did not meet the designed sample size of 273? From 256 with a response rate of 93.4%, which means only 239 patients have response, it is less than the target sample size n=246. Please discuss if it will it affect the results.

***Response #2:*** *We appreciate for the comment. The study achieved beyond the minimum sample size required in planning stage of the study. We interviewed 256 patients while the minimum sample size required was 246. The study achieved 93.8% response rate, which could be obtained by dividing the actual response divided by the plan (256/273 = 93.8%). The actual non-response rate is below the contingency plan of 10%. Thus, the number of patients included in the study are adequate and have not any negative effect on the results.*

**Comment #3:** In Table 4, what does COR or AOR stand for? Please add notes corresponding to them. It is also not clear about the QoL. Is it functional QoL, symptom QoL, or global QoL?

***Response #3:*** *Thank you for the comment. We revised the manuscript as per the reviewer comment. There is no single definition of QoL but scholars define it in different ways; it is “the subjective evaluation of the good and satisfactory character of life as a whole”[**1**]. The WHO defines QoL as “an individual’s perception of their position in life in the context of the culture and value systems in which they live and in relation to their goals, expectations, standards, and concerns*[2]*”.*

- *Symptom QoL relates to the individual's experience of symptoms or discomfort associated with breast cancer.*
- *Global health (QoL) reflects an overall assessment of an individual's satisfaction and happiness with their life as a whole.*
- *Functional QoL: refers to the individual's ability to perform daily activities and tasks related to their physical, emotional, and social well-being, despite the challenges posed by the disease and its treatment.*

*The EORTC QLQ-C30 is a tool used to address quality of life issues to all cancer type patients and it is composed of nine multi-item scales and six single items. The multi-item scales include five functioning scales (physical, role, cognitive, emotional, and social), a global health status (QoL) scale, and three symptom scales (fatigue, pain, and nausea/vomiting). The six single items included dyspnea, insomnia, appetite loss, constipation, diarrhea, and financial difficulties [**3**]. The EORTC QLQ-BR23 is unique to breast cancer patients and it is composed of four functional scales (future perspective, body image, sexual function, and sexual enjoyment) and four symptom scales (systemic therapy side effect, arm symptom, breast symptom, financial difficulties, and upset by hair loss).*

***Reference***

1.	van Knippenberg FC, d.H.J., *Measuring the quality of life of cancer patients: psychometric properties of instruments.* J Clin Epidemiol, 1988. **41**(1043-1053).

2.	Group, W., *Study protocol for the World Health Organization project to develop a Quality of Life assessment instrument (WHOQOL).* Quality of life Research, 1993. **2**(2): p. 153-159.

3.	Aaronson NK., A.S., Bergman B, et al. , *The European Organisation for Research and Treatment of Cancer QLQ-C30: A quality-of-life instrument for use in international clinical trials in oncology.* Journal of the National Cancer Institute, 1993. **85**: p. 365-376.
