# Supplementary material for: Quality of life of breast cancer patients in Amhara region, Ethiopia: A cross-sectional study
Source: PLoS One. 2024 Jun 27;19(6):e0305263. doi: 10.1371/journal.pone.0305263 (PMC11210875; doi:10.1371/journal.pone.0305263)
Supplement: Attachment — Submitted filename: Response to reviewer.docx [file pone.0305263.s007.docx]

**Response to reviewer**

**Response to reviewer #3**

**Comment #**1: For the QoL, it sounds that Table 4 used QLQ-BR23 to determine subject’s QoL outcome as good or poor QoL. Since QLQ-BR23 includes both functional and symptom items, please provide details about how to assign the dichotomous outcome to each subject. E.g. for one subject, if her average functional scale (>=75) is good, but average symptom scale (>=25) is poor, will this subject’s QoL be considered as poor?

***Response #1:*** *We appreciate for the comment. The EORTC QLQ-BR23 is unique to breast cancer patients and it is composed of four functional scales (future perspective, body image, sexual function, and sexual enjoyment) and four symptom scales (systemic therapy side effect, arm symptom, breast symptom, and upset by hair loss). The functional scale assesses various aspects related to the patient’s well-being, and the symptom scale evaluates symptoms experienced by the patient.* *The breast cancer tools incorporates five multi-item scales to assess systemic therapy side effects, arm symptoms, breast symptoms, body image and sexual functioning. In addition, single items assess sexual enjoyment, hair loss and future perspective [**1**].*

*The quality of life (QoL) of breast cancer patients had two global health status questions, with a modified 7-point linear analog scale ranging from 1 “very poor” to 7 “excellent”. For one subject, if her average functional scale (>=75) is good, and average symptom scale (>=25) is poor, will this subject’s QoL might be good or poor. The patients QoL determined by the result of global health status scores, without considering the functional and symptoms scale results (See below Table 1).*

**Table 1: scoring of EORTC QLQ-C30 and QLQ-BR23**

| EORTC QLQ-C30 and QLQ-BR23 | *Scale* | *Number of items* | I*tem number* | *Item range* |
| --- | --- | --- | --- | --- |
| QoL | Global health status/QoL | *2* | 29, 30 | *6* |
| Functional scales | *Physical functioning* | *5* | 1 to 5 | *3* |
|  | *Role functioning* | *2* | 6, 7 | *3* |
|  | *Emotional functioning* | *4* | 21 to 24 | *3* |
|  | *Cognitive functioning* | *2* | 20, 25 | *3* |
|  | *Social functioning* | *2* | 26, 27 | *3* |
| Symptom scale or items | *Fatigue* | *3* | 10, 12, 18 | *3* |
|  | *Nausea and vomiting* | *2* | 14, 15 | *3* |
|  | *Pain* | *2* | 9, 19 | *3* |
|  | *Dyspnea* | *1* | 8 | *3* |
|  | *Insomnia* | *1* | 11 | *3* |
|  | *Appetite loss* | *1* | 13 | *3* |
|  | *Constipation* | *1* | 16 | *3* |
|  | *Diarrhea* | *1* | 17 | *3* |
|  | *Financial difficulties* | *1* | 28 | *3* |
| *Functional scales* | *Body image* | *4* | *9-12* | *3* |
|  | *Sexual function* | *21* | *14, 15* | *3* |
|  | *Sexual enjoyment* | *1* | *16* | *3* |
|  | *Future perspective* | *1* | *13* | *3* |
| *Symptom scales/items* | *Systemic therapy side effects* | *7* | *1-4, 6-8* | *3* |
|  | *Breast symptom* | *4* | *20-23* | *3* |
|  | *Arm symptom* | *3* | *17-19* | *3* |
|  | *Upset by hair loss* | *1* | *5* | *3* |

*“Item range” is the difference between the maximum and the minimum response to individual items.*

***Reference***

1.	Aaronson NK., A.S., Bergman B, et al. , *The European Organisation for Research and Treatment of Cancer QLQ-C30: A quality-of-life instrument for use in international clinical trials in oncology.* Journal of the National Cancer Institute, 1993. **85**: p. 365-376.
